# HbA1c Levels Are Associated with Chronic Kidney Disease in a Non-Diabetic Adult Population: A Nationwide Survey (KNHANES 2011–2013)

**DOI:** 10.1371/journal.pone.0145827

**Published:** 2015-12-30

**Authors:** Seok Hui Kang, Da Jung Jung, Eun Woo Choi, Kyu Hyang Cho, Jong Won Park, Jun Young Do

**Affiliations:** 1 Division of Nephrology, Department of Internal Medicine, Yeungnam University Hospital, Daegu, Republic of Korea; 2 Department of Otorhinolaryngology-Head and Neck Surgery, School of Medicine, Kyungpook National University Hospital, Daegu, Republic of Korea; Kaohsiung Chang Gung Memorial Hospital, TAIWAN

## Abstract

**Background:**

Many studies have reported an association between glycated hemoglobin A1c (HbA1c) and metabolic syndrome (MetS) in non-diabetes patients. Each component of MetS is in fact related to chronic kidney disease (CKD) incidence and progression. Therefore, HbA1c in non-diabetic mellitus (DM) may be intrinsically associated with the prevalence of CKD. The hypothesis of the present study was that high HbA1c in non-DM patients is associated with CKD.

**Patients and Methods:**

The total number of participants in this study was 24,594. The participants were divided into three groups according to their HbA1c levels: a Low group (<5.7% or <39 mmol/mol), a Middle group (5.7–6.0% or 39–42 mmol/mol), and a High group (>6.0% or >42 mmol/mol). The estimated glomerular filtration rate (eGFR) was calculated using the Chronic Kidney Disease Epidemiology Collaboration equation.

**Results:**

The number of participants allocated to the Low, Middle, and High groups was 8,651, 4,634, and 1,387, respectively. Linear regression analyses were performed to evaluate the association between variables. Standardized β ± standard error was 0.25 ± 0.22 for waist circumference, 0.44 ± 0.20 for fasting glucose, –0.14 ± 0.30 for high-density lipoprotein cholesterol levels, 0.15 ± 2.31 for triglyceride levels, 0.21 ± 0.00 for systolic blood pressure, 0.10 ± 0.00 for diastolic blood pressure, and –0.22 ± 0.42 for eGFR (*P* < 0.001 for all variables). eGFR in non-diabetes participants was inversely associated with the HbA1c level, where eGFR decreased as HbA1c levels increased. Standardized βs were –0.04 ± 0.42 in multivariable analysis (*P* < 0.001). The proportion of participants with only MetS, only CKD, or both MetS and CKD was higher in the High group than in the Low and Middle groups.

**Conclusion:**

High HbA1c in non-DM patients may be associated with CKD. Renal function in patients with high HbA1c levels may need to be monitored.

## Background

Chronic kidney disease (CKD) is a widely recognized public health issue and associated with high morbidity and mortality when compared to the non-CKD population [1,2]. The United States Real Data System 2014 Annual Data Report showed that CKD occurs in approximately 13.6% of the general population [3]. Indeed, the prevalence of CKD appears to be rising rapidly with increased life expectancy. Overall Medicare expenditures for CKD were $44,581 million in 2012 [3]. Screening for and effective monitoring of CKD are essential for increasing patient quality of life and decreasing the public health burden.

Glycated hemoglobin (HbA1c) is an important indicator for long-term glucose control and has recently been recommended for use in the diagnosis of diabetes mellitus (DM) by the American Diabetes Association (ADA) [4]. However, the use of HbA1c for identifying pre-diabetes is a controversial topic [5]. In 2015, the ADA suggested that an HbA1c of 5.7–6.4% (39–46 mmol/mol) is reasonable for the diagnosis of pre-diabetes and that patients with HbA1c > 6.0% (>42 mmol/mol) should be considered to be at very high risk for DM [4]. Although the clinical significance of HbA1c as a surrogate marker of metabolic syndrome (MetS) has not yet been fully examined, many studies have reported an association between HbA1c and MetS in non-DM patients [6–8]. Each component of MetS is in fact related to CKD incidence and progression [9]. Therefore, HbA1c in non-DM may be intrinsically associated with the prevalence of CKD. The aim of the present study was to evaluate the clinical association between HbA1c and CKD in non-DM patients. The hypothesis of the present study was that high HbA1c in non-DM patients is associated with CKD.

## Patients and Methods

### Study population

Data from the Korean National Health and Nutrition Examination Survey (KNHANES 2011–2013) were used for this analysis. The KNHANES is a nationwide, multi-stage, stratified survey of a representative sample of the South Korean population and is conducted by the Korea Centers for Disease Control and Prevention. The total number of participants from KNHANES analyzed in this study was 24,594. Participants were excluded from the present study based on the following criteria: data could not be provided for HbA1c (n = 2,350) or renal function (n = 2) or participants were younger than 18 years of age (n = 5,385) or had DM (defined as a self-reported history of a DM diagnosis, a fasting glucose level of ≥126 mg/dL, or HbA1c ≥ 6.5% (≥48 mmol/mol; n = 2,185). As a result, 14,672 participants were ultimately included in this study. Ethical approval for this study was obtained from the institutional review board of Yeungnam University Hospital (2015-04-004). The board waived the need for informed consent, as the subjects’ records and information were anonymized and de-identified prior to analysis.

### Study variables

Clinical and laboratory data collected during clinical examination included the following: age, sex, serum creatinine (mg/dL), body mass index (BMI, kg/m^2^), waist circumference (WC, cm), HbA1c (%, mmol/mol), fasting blood glucose (mg/dL), total cholesterol (mg/dL), high-density lipoprotein (HDL) cholesterol levels (mg/dL), triglyceride levels (mg/dL), systolic blood pressure (mmHg), diastolic blood pressure (mmHg), smoking status, alcohol intake, and levels of physical activity.

HbA1c levels were measured using a high performance liquid chromatography system (HLC-723G7; Tosoh Co., Tokyo, Japan). In the present study, the participants were divided into three groups according to their HbA1c levels: a Low group (<5.7% or <39 mmol/mol), a Middle group (5.7–6.0% or 39–42 mmol/mol), and a High group (>6.0% or >42 mmol/mol). Serum creatinine levels were measured using a Hitachi Automatic Analyzer (alkaline picrate, Jaffé kinetic). The estimated glomerular filtration rate (eGFR) was calculated using the Chronic Kidney Disease Epidemiology Collaboration (CKD-EPI) equation [10]. CKD was defined as an eGFR <60 mL/min/1.73 m^2^. Urine albumin level was measured from random samples using a turbidimetric immunoassay (Hitachi Automatic Analyzer 7600, Hitachi). Urine creatinine level was measured using a colorimetric method (Hitachi Automatic Analyzer 7600, Hitachi). Urine albumin and creatinine concentrations were measured in the same laboratory for all surveys. The inter-assay coefficient of variation for all laboratory work was consistenly low (<3.1%). The urine albumin-creatinine ratio (UACR) was calculated in mg per g of creatinine (mg/g). Albuminuria was defined as UACR ≥30 mg/g.

Patients were classified according to smoking status as current smokers, ex-smokers, or non-smokers. Alcohol intake was defined using the Korean version of ‘standard drinking’ based on the WHO classification [11,12]. Alcohol intake was classified into 3 categories: abstinence (no consumption of alcohol within the last year); moderate drinking (women: 0.1–19.99 g pure alcohol/day; men: 0.1–39.99 g pure alcohol/day), and heavy drinking (women: ≥20 g pure alcohol/day; men: ≥40 g pure alcohol/day). Physical activity was assessed by the presence of exercise. The presence of exercise was defined as moderate activity for more than 30 min/day, for 5 days/week or intense activity for more than 20 min/day, for 3 days/week, or walking more than 30 min/day, for 5 days/week. Coronary artery disease (CAD) was defined as a self-reported history of angina or myocardial infarction. Cerebrovascular accident (CVA) was defined as a self-reported history of stroke.

MetS was defined according to the Adult Treatment Panel III criteria using the modified cutoff values for Asian populations as suggested by the Asia-Pacific guidelines [13,14]. Briefly, elevated blood glucose was defined as a fasting blood glucose level ≥100 mg/dL or a self-reported history of DM. Elevated blood pressure was defined as a systolic or diastolic blood pressure ≥130/85 mmHg and a self-reported history of hypertension. A low HDL cholesterol level was defined as <40 mg/dL in men and <50 mg/dL in women. Elevated triglyceride levels were defined as a serum triglyceride level of ≥150 mg/dL. Abdominal obesity was defined as a WC >90 cm in men and >80 cm in women. MetS was defined as the presence of ≥3 components of MetS.

### Statistical analyses

The data were analyzed using the Statistical package for the Social Sciences software package (SPSS v.21, Chicago, IL., USA). Categorical variables were expressed as both counts and percentages. Continuous variables were expressed as the mean ± standard deviation (SD). UACR was a non-parametric variable expressed as a median (95% CI), and was compared using the Kruskal-Wallis test. The Pearson’s χ^2^ or Fisher’s exact test was used to analyze categorical variables. For continuous variables, means were compared using a one-way analysis of variance. Correlations were analyzed in order to assess the strength of the relationship between continuous variables. Linear regression analysis was performed to assess independent predictors of eGFR or number of MetS components. Variance inflation factor was used to identify multicollinearity for the multivariable linear regression model. Variance inflation factor greater than 10 was not accepted. Logistic regression analyses were used for estimating the odds ratios (OR) and 95% confidence intervals (CI), which were then applied towards determining the relationship between HbA1c and CKD or MetS.

Confounders were defined by their likelihood of preceding or contributing to the development of MetS or CKD. The selection of confounder was based on previous literatures [15,16]. For MetS, the covariates were HbA1c, age, sex, BMI, alcohol intake, smoking status, and physical activity. For CKD, the covariates were HbA1c, age, sex, BMI, alcohol intake, smoking status, physical activity, CAD, CVA, WC, HDL cholesterol levels, triglyceride levels, systolic blood pressure, and diastolic blood pressure. Discrimination–which is the ability of the model to differentiate between participants who have CKD or MetS and those who do not–was examined using the area under the receiver operating characteristic (AUROC) curve. AUROC analysis was also performed in order to calculate cutoff values, sensitivity, and specificity. Optimal cutoff risk point was defined as the maximum Youden index in the AUROC. The AUROC was calculated using the MedCalc software package (v.11.6.1.0, MedCalc, Mariakerke, Belgium). We also calculated the integrated discrimination improvement (IDI) and the net reclassification improvement (NRI) with a category-free option among models, following the methodology of Penica et al. [17,18]. A restricted cubic spline curve was used to evaluate non-linear relationships between the HbA1c level and CKD, which was adjusted for age and sex. The restricted cubic spline curve was plotted using statistical software SAS version 9.4 (SAS Campus Drive, Cary, NC, USA). A *P*-value less than 0.05 was considered statistically significant.

## Results

### Clinical characteristics of participants

The number of participants allocated to the Low, Middle, and High groups was 8,651, 4,634, and 1,387, respectively (Table 1). Age, BMI, WC, eGFR, total cholesterol, fasting blood glucose, triglyceride levels, and systolic and diastolic blood pressure were higher in the High group than either the Low or Middle group.

**Table 1:** Clinical characteristics of participants by HbA1c level.

	Low (n = 8,651)	Middle (n = 3,865)	High (n = 2,156)	*P*-value*
Age (years)	43.2 ± 15.6	53.2 ± 15.4	59.7 ± 12.6	<0.001
Sex (male, %)	3,608 (41.7%)	2,037 (44.0%)	563 (40.6%)	0.017
HbA1c (%, mmol/mol)	5.35 ± 0.22, 35 ± 2	5.82 ± 0.11, 40 ± 1	6.20 ± 0.11, 44 ± 1	<0.001
Body mass index (kg/m^2^)	23.0 ± 3.2	24.0 ± 3.3	24.9 ± 3.3	<0.001
Creatinine (mg/dL)	0.82 ± 0.19	0.84 ± 0.18	0.86 ± 0.37	<0.001
Waist circumference (cm)	78.3 ± 9.5	81.9 ± 9.3	84.6 ± 9.2	<0.001
Total cholesterol (mg/dL)	184.0 ± 33.7	196.8 ± 36.0	199.4 ± 39.8	<0.001
Fasting blood glucose (mg/dL)	90.1 ± 7.9	95.3 ± 8.9	102.1 ± 10.2	<0.001
Triglyceride (mg/dL)	115.3 ± 91.3	138.6 ± 105.1	152.4 ± 104.5	<0.001
High density lipoprotein (mg/dL)	54.6 ± 12.9	51.9 ± 11.9	49.6 ± 11.7	<0.001
Systolic blood pressure (mmHg)	114.9 ± 15.8	120.0 ± 16.8	124.6 ± 17.0	<0.001
Diastolic blood pressure (mmHg)	74.8 ± 10.4	76.3 ± 10.3	77.0 ± 10.4	<0.001
Physical activity (%)	3897 (45.0%)	1,993 (45.2%)	543 (40.9%)	<0.001
Coronary artery disease (%)	72 (0.8%)	113 (2.4%)	70 (5.0%)	<0.001
Cerebrovascular accident (%)	65 (0.8%)	87 (1.9%)	45 (3.2%)	<0.001
Alcohol intake				<0.001
Abstinence	1866 (21.6%)	1,334 (28.8%)	505 (36.4%)	
Moderate drinking	6071 (70.2%)	2,929 (63.2%)	784 (56.5%)	
Heavy drinking	382 (4.4%)	147 (3.2%)	39 (2.8%)	
Unknown	332 (3.8%)	224 (4.8%)	59 (4.3%)	
Smoking				0.004
Non-smoker	5190 (60.0%)	2,616 (56.5%)	806 (58.1%)	
Ex-smoker	1490 (17.2%)	897 (19.4%)	265 (19.1%)	
Current smoker	1653 (19.1%)	902 (19.5%)	257 (18.5%)	
Unknown	318 (3.7%)	219 (4.7%)	59 (4.3%)	
eGFR (mL/min/1.73 m^2^)	96.6 ± 18.0	91.0 ± 17.0	87.0 ± 17.2	<0.001

Data are expressed as numbers (percentages) for categorical variables and mean ± standard deviations for continuous variables.

*P values were tested by one-way analysis of variance for continuous variables and Pearson χ^2^ test or Fisher exact test for the categorical variables.

The proportion of participants with only MetS in the Low, Middle, and High groups was 9.1%, 20.4%, and 33.9%, respectively (*P* < 0.001), whereas the proportion of participants with only CKD in the Low, Middle, and High groups was 0.9%, 2.0%, and 3.5%, respectively (*P* < 0.001). The proportion of participants with both MetS and CKD in the Low, Middle, and High groups was 0.2%, 0.7%, and 2.0%, respectively (*P* < 0.001). The proportion of participants with only MetS, only CKD, or both MetS and CKD was higher in the High group than in the Low and Middle groups.

### Association between HbA1c level and MetS or CKD

We performed univariate linear regression analyses to evaluate the association between HbA1c and each MetS components. Standardized β ± standard error was 0.25 ± 0.22 for WC, 0.44 ± 0.20 for fasting glucose,–0.14 ± 0.30 for HDL cholesterol levels, 0.15 ± 2.31 for triglyceride levels, 0.21 ± 0.00 for systolic blood pressure, and 0.10 ± 0.00 for diastolic blood pressure (*P* < 0.001 for all variables). There were positive associations between HbA1c levels and WC, fasting glucose, triglyceride levels, systolic blood pressure, and diastolic blood pressure, and negative association between HbA1c levels and HDL cholesterol levels. In addition, HbA1c in non-diabetes participants was associated with the number of MetS components observed (S1 Table). Numbers of MetS components increased in accordance with increased HbA1c levels.

Univariate and multivariable linear regression analyses were also performed to evaluate the association between HbA1c level and eGFR (S1 Table). eGFR in non-diabetes participants was inversely associated with the HbA1c level, where eGFR decreased as HbA1c levels increased.

Logistic regression showed that the OR for only MetS with a 1% (11 mmol/mol) increase in HbA1c was 7.53 (95% CI, 6.51–8.70) in univariate analysis and 3.38 (95% CI, 2.85–4.00) in multivariable analysis (S2 Table). The OR for only CKD with a 1% (11 mmol/mol) increase in HbA1c was 9.32 (95% CI, 6.16–14.11) in univariate analysis and 2.13 (95% CI, 1.33–3.40) in multivariable analysis. The OR for both MetS and CKD with a 1% (11 mmol/mol) increase in the level of HbA1c was 21.49 (95% CI, 10.86–42.52) on univariate analysis and 4.12 (95% CI, 1.80–9.39) on multivariable analysis. A restricted cubic spline curve was plotted, with 5.6% (38 mmol/mol) as the median HbA1c level, and it was adjusted for age and sex (S1 Fig). A high HbA1c level was associated with increased OR for CKD.

To estimate the incremental value of HbA1c level to predict only MetS, only CKD or both MetS and CKD, we compared the probabilities of events and nonevents of models using relative IDI and category-free NRI (S3 Table). The IDI of adding HbA1c level to the multivariable model improved significantly. The addition of HbA1c to multivariable models resulted in a significant improvement of the category-free NRI.

**Fig 1:**
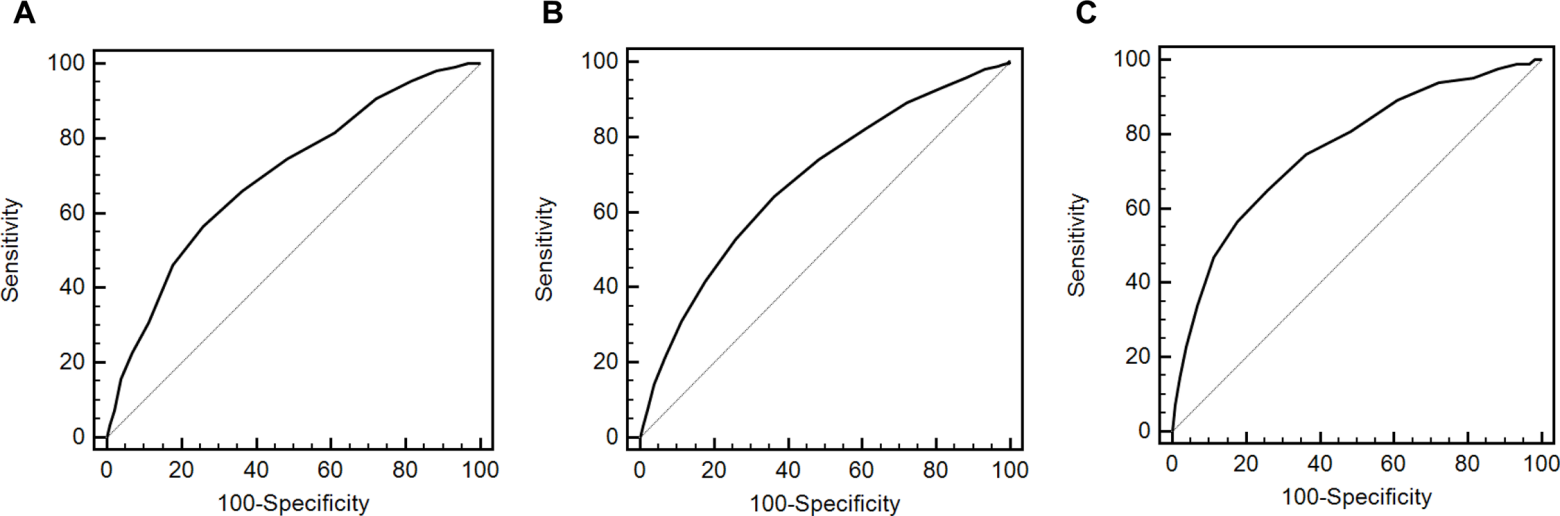
Receiver operating characteristic curves of HbA1c for the prediction of metabolic syndrome or chronic kidney disease. A. Only metabolic syndrome. B. Only chronic kidney disease. C. Both metabolic syndrome and chronic kidney disease.

The AUROC value of HbA1c was 0.700 (95% CI, 0.692–0.708) for only MetS, 0.685 (95% CI, 0.678–0.693) for only CKD, and 0.760 (95% CI, 0.752–0.768) for both MetS and CKD (*P* < 0.001). The cutoff value was >5.7% **(>**39 mmol/mol) for only MetS, >5.6% (>38 mmol/mol) for only CKD, and >5.7% (>39 mmol/mol) for both MetS and CKD (Fig 1). Sensitivity and specificity for predicting only MetS were 56.7% and 74.2%, respectively. Those for predicting only CKD were 64.2% and 63.8%, respectively, while those for predicting both MetS and CKD were 65.1% and 74.2%, respectively.

### Association between HbA1c level and UACR

In participants with eGFR ≥60 mL/min/1.73 m^2^, the correlation coefficient between UACR and HbA1c was 0.043 (*P* < 0.001). UACR in the Low, Middle, and High groups was 9.2 (95% CI, 8.1–10.2), 11.5 (95% CI, 9.9–13.1), and 15.9 (95% CI, 12.6–19.1), respectively (*P* < 0.001). The portion of participants with albuminuria in the Low, Middle, and High groups was 329 (4.3%), 239 (5.6%), and 104 (8.2%), respectively (*P* < 0.001).

## Discussion

In the present study, a clear association was observed between HbA1c and MetS in non-DM Asian patients, which is in line with numerous other studies that have shown an association between these two variables [6–9,19–23]. Studies aiming to investigate this association should exclude patients with DM as this condition is a critical confounding factor for the prevalence of MetS and certain studies have reported no exclusion of patients with HbA1c ≥ 6.5% (≥48 mmol/mol) [21,23]. While a few previous studies did exclude DM patients with HbA1c ≥ 6.5% (≥48 mmol/mol), the majority of these were single-center studies with a possibility of selection bias [8,21,23]. The present study analyzed a nationwide, multi-stage, stratified survey of a representative sample of the South Korean population and excluded patients with HbA1c ≥ 6.5% (≥48 mmol/mol). Results were adjusted for variable confounders and revealed that HbA1c in non-DM patients is associated with the number of MetS components. The linear regression analyses did show an association between HbA1c and each component of MetS as continuous variables.

The present study showed an association between the HbA1c level and CKD with or without MetS. The associations between insulin resistance and CKD are very complex and not clear. Previous studies have shown that each component of MetS is associated with development and progression of CKD. Among the components of MetS, insulin resistance may be the most important related etiological factor for CKD [24]. HbA1c is an indicator predicting insulin resistance. High HbA1c level in pre-diabetes is associated with insulin resistance or metabolic syndrome, which can lead to development and progression of CKD. Our results suggest that high HbA1c is mainly associated with insulin resistance, which may result in development of CKD. However, CKD results in interference with the intracellular signaling pathway initiated by insulin, which results in insulin resistance [25].

The literature has shown conflict results concerning an association between HbA1c and CKD. Certain studies have shown that HbA1c is associated with development of CKD in non-DM patients [26–29]. Gerstein et al. conducted a prospective study with an average 4.5-year follow-up and successfully showed that HbA1c is associated with development of overt nephropathy defined by albuminuria or proteinuria [26]. Zhang et al. evaluated a cross-sectional study using a German cohort and showed an association between HbA1c and eGFR or CKD defined as eGFR < 60 mL/min/1.73 m^2^ [27]. Although DM was adjusted for in multivariable analysis, this study did include DM patients. A study by Plantinga et al. enrolled non-DM patients using data from the USA; however, this group evaluated the association between CKD and pre-diabetic status classified by fasting glucose level [28]. In contrast to the afore-mentioned studies, there have been reports that no association exists between the two variables if variable cardiovascular risk factors are adjusted for [30–33]. Selvin et al. observed no significant difference in the development of CKD in patients with HbA1c 5.7–6.5% (39–48 mmol/mol) when compared to patients with HbA1c <5.7% (<39 mmol/mol) [31]. However, that study did not include HbA1c as a diagnostic criterion for DM and could possibly include DM patients. To the best of our knowledge, this is the first study to evaluate the association between HbA1c and CKD in an Asian population. The results of the present study do show an association between HbA1c and eGFR as a continuous variable for renal function or CKD as a categorical variable. In addition, we calculated IDI and NRI as more advanced prediction analyses; these analyses showed that in comparison with multivariable models using only traditional risk factors, the addition of HbA1c to multivariable models improved both IDI and NRI. Unfortunately, cross-sectional study such as this cannot evaluate the causal relationship among these variables. Further prospective studies are needed to identify the causality between two variables.

The present study showed the association between HbA1c and albuminuria as a surrogate marker for early CKD. In participants with eGFR ≥60 mL/min/1.73 m^2^, UACR and the proportion of participants with albuminuria increased as HbA1c increased. Previous studies demonstrated that HbA1c level is associated with albuminuria in participants with DM [34–39]. Poor glycemic control in DM plays a key role in rapid progression to diabetic nephropathy, which is caused by variable hemodynamic, metabolic, or endothelial dysfunction [40]. Many previous studies have demonstrated pathophysiology or factors associated with progression to albuminuria in DM, but there have been few studies regarding the association between HbA1c and albuminuria in non-DM participants. The present study reveals that high-normal HbA1c levels previously considered to be in the normal range may be associated with albuminuria and may function as a marker for early CKD in non-DM participants.

The pathophysiology of the association between CKD and HbA1c in non-DM participants may be the same as that in high-glucose DM participants. This may ultimately result in subclinical or clinical atherosclerosis in various vessels. Glycemic control is a well-known risk factor for the development of atherosclerosis in DM participants. Previous studies also showed a positive association between prediabetes and atherosclerosis as measured by carotid intimal thickness, subclinical myocardial damage, or coronary artery calcium [41–43]. These pathologic changes can develop in the renal vasculature, which results in CKD with albuminuria.

Very low HbA1c level may be associated with malnutrition, inflammation, and atherosclerosis. However, in our study, a spline curve showed that low HbA1c level is not associated with CKD compared to median HbA1c level. Two factors may be associated with this discordance. First, malnutrition combined with low HbA1c level is common in participants with severe comorbidities, such as advanced cancer or end-stage renal disease, compared with the general population. Participants enrolled in our study may have been healthier than other selected populations who visited hospitals, which could have resulted in selection bias. Second, HbA1c may be used as a nutritional marker, but it mainly reflects glucose intake. The numbers of participants with total cholesterol < 100 mg/dL as another marker of malnutrition were 6, 7, and 0 in the Low, Middle, and High groups, respectively. There were few participants with malnutrition defined by total cholesterol level in our study.

This study has a number of limitations. First, it is a retrospective cross-sectional design and therefore cannot establish causality between two variables. Second, the available data did not include post-prandial blood glucose levels as a criterion for DM and a small number of DM patients could therefore have included. However, all participants have HbA1c < 6.5% (<48 mmol/mol) and a fasting blood glucose <126 mg/dL. Third, KDIGO guidelines define CKD as eGFR < 60 mL/min/1.73 m^2^ for >3 months [44]. In our study, CKD was defined using a single serum creatinine or single spot urine sample. However, the effect of these limitations will be reduced by the strength of a nation-wide representative sample.

In conclusion, high HbA1c in non-DM patients may be associated with CKD. Renal function in patients with high HbA1c levels may need to be monitored.

## Supporting Information

S1 Fig Adjusted restricted cubic spline curve showing odds ratio and 95% confidence interval (dashed line) for chronic kidney disease associated with HbA1c level (median value = 5.6% or 38 mmol/mol). Spline curve was adjusted for age and sex.
(TIF)

S1 Table Linear regression analyses for the number of metabolic syndrome components or estimated glomerular filtration rate according to HbA1c level. (DOCX)

S2 Table Logistic regression analyses for metabolic syndrome or chronic kidney disease according to HbA1c level. (DOCX)

S3 Table AUCs, IDI, and NRI for multivariable models with or without HbA1c level. (DOCX)
