# Supplementary figures and images for: HbA1c Levels Are Associated with Chronic Kidney Disease in a Non-Diabetic Adult Population: A Nationwide Survey (KNHANES 2011–2013)
Source: PLoS One. 2015 Dec 30;10(12):e0145827. doi: 10.1371/journal.pone.0145827 (PMC4696727; doi:10.1371/journal.pone.0145827)

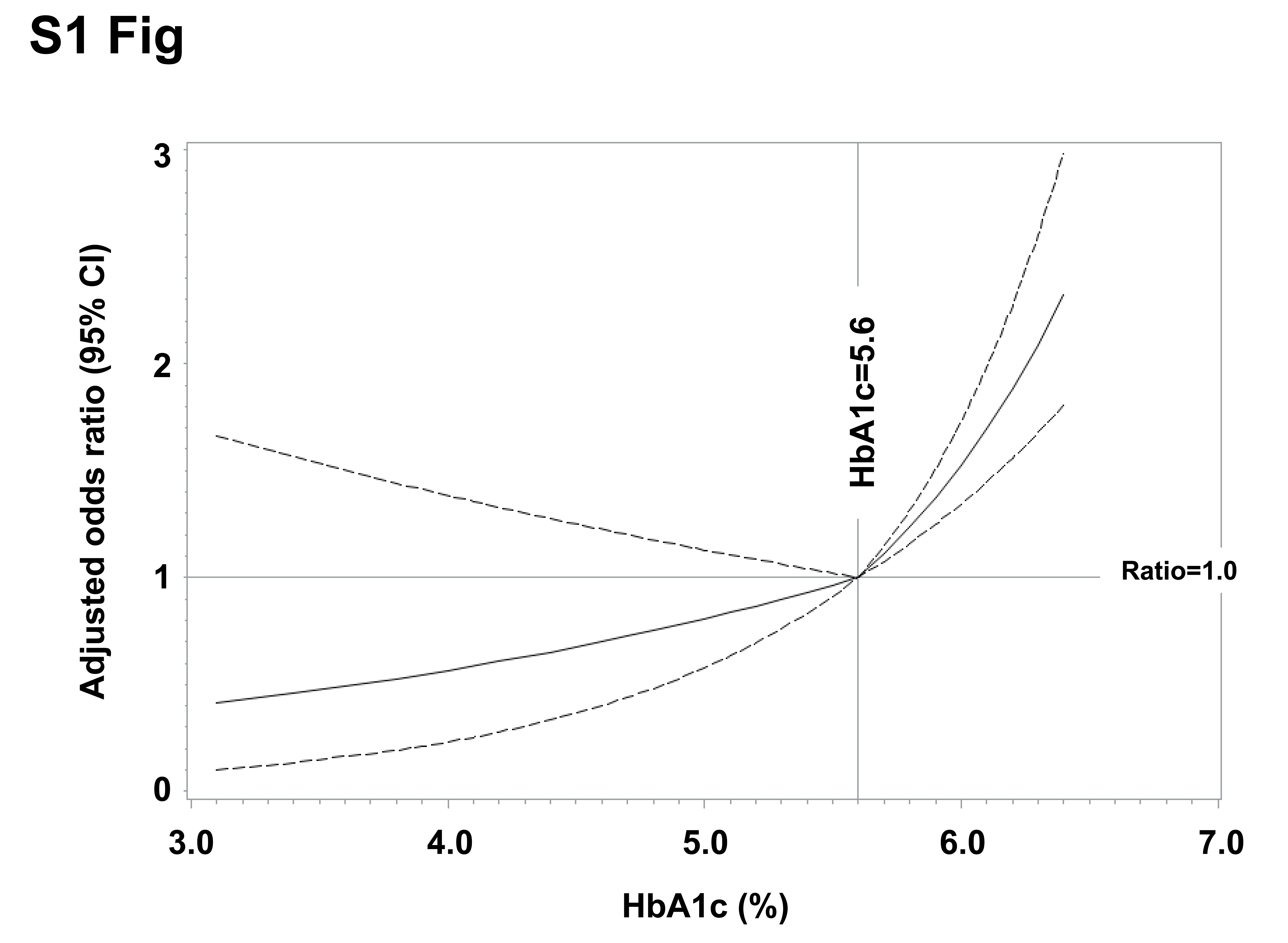

Supplement: S1 Fig — Spline curve was adjusted for age and sex. (TIF) [file pone.0145827.s001.tif]
