# Supplementary material for: HbA1c Levels Are Associated with Chronic Kidney Disease in a Non-Diabetic Adult Population: A Nationwide Survey (KNHANES 2011–2013)
Source: PLoS One. 2015 Dec 30;10(12):e0145827. doi: 10.1371/journal.pone.0145827 (PMC4696727; doi:10.1371/journal.pone.0145827)
Supplement: S1 Table — (DOCX) [file pone.0145827.s002.docx]

|  | **Univariate** | |  | **Multivariable** | |  | |  |
| --- | --- | --- | --- | --- | --- | --- | --- | --- |
|  | **Standardized β ± SE** | ***P*-value** |  | **Standardized β ± SE** | ***P*-value*** | | **VIF** | |
| Dep: number of MetS components |  |  |  |  |  | |  | |
| HbA1c level | 0.34 ± 0.03 | <0.001 |  | 0.15 ± 0.03 | <0.001 | | 1.234 | |
| Age | 0.34 ± 0.00 | <0.001 |  | 0.24 ± 0.00 | <0.001 | | 1.254 | |
| Sex | –0.13 ± 0.02 | <0.001 |  | –0.04 ± 0.02 | <0.001 | | 1.706 | |
| Body mass index | 0.54 ± 0.00 | <0.001 |  | 0.48 ± 0.00 | <0.001 | | 1.070 | |
| Alcohol intake | –0.02 ± 0.02 | 0.015 |  | 0.04 ± 0.02 | <0.001 | | 1.169 | |
| Smoking status | 0.10 ± 0.01 | <0.001 |  | 0.04 ± 0.01 | <0.001 | | 1.727 | |
| Physical activity | 0.05 ± 0.02 | <0.001 |  | –0.03 ± 0.02 | <0.001 | | 1.013 | |
| Dep: eGFR |  |  |  |  |  | |  | |
| HbA1c level | –0.22 ± 0.42 | <0.001 |  | –0.04 ± 0.42 | <0.001 | | 1.245 | |
| Age | –0.45 ± 0.01 | <0.001 |  | –0.46 ± 0.01 | <0.001 | | 1.719 | |
| Sex | 0.13 ± 0.30 | <0.001 |  | 0.15 ± 0.38 | <0.001 | | 2.016 | |
| Body mass index | –0.12 ± 0.04 | <0.001 |  | –0.14 ± 0.08 | <0.001 | | 4.335 | |
| Alcohol intake | 0.08 ± 0.30 | <0.001 |  | –0.01 ± 0.29 | 0.161 | | 1.203 | |
| Smoking status | –0.05 ± 0.19 | <0.001 |  | 0.02 ± 0.22 | 0.025 | | 1.748 | |
| Physical activity | 0.00 ± 0.30 | 0.774 |  | –0.02 ± 0.27 | 0.043 | | 1.022 | |
| Waist circumference | –0.17 ± 0.02 | <0.001 |  | 0.13 ± 0.03 | <0.001 | | 5.034 | |
| HDL cholesterol level | 0.14 ± 0.01 | <0.001 |  | 0.05 ± 0.01 | <0.001 | | 1.305 | |
| Triglyceride level | –0.06 ± 0.00 | <0.001 |  | 0.04 ± 0.00 | <0.001 | | 1.277 | |
| Systolic blood pressure | –0.22 ± 0.01 | <0.001 |  | 0.07 ± 0.01 | <0.001 | | 2.368 | |
| Diastolic blood pressure | –0.13 ± 0.01 | <0.001 |  | –0.09 ± 0.02 | <0.001 | | 2.021 | |
| Coronary artery disease | –0.10 ± 1.12 | <0.001 |  | –0.03 ± 1.00 | <0.001 | | 1.028 | |
| Cerebrovascular accident | –0.08 ± 1.28 | <0.001 |  | –0.02 ± 1.14 | 0.010 | | 1.020 | |

*Multivariable analysis for number of MetS components was performed using HbA1c level, age, sex, body mass index, alcohol intake, smoking status, and physical activity. Multivariable analysis for eGFR was performed using HbA1c level, age, sex, body mass index, alcohol intake, smoking status, physical activity, waist circumference, HDL cholesterol level, triglyceride level, systolic blood pressure, diastolic blood pressure, coronary artery disease, and cerebrovascular accident.

Abbreviations: SE, standard error; VIF, variance inflation factor; Dep, dependent variable; MetS, metabolic syndrome; eGFR, estimated glomerular filtration rate; HDL, high-density lipoprotein.
