# Supplementary material for: HbA1c Levels Are Associated with Chronic Kidney Disease in a Non-Diabetic Adult Population: A Nationwide Survey (KNHANES 2011–2013)
Source: PLoS One. 2015 Dec 30;10(12):e0145827. doi: 10.1371/journal.pone.0145827 (PMC4696727; doi:10.1371/journal.pone.0145827)
Supplement: S2 Table — (DOCX) [file pone.0145827.s003.docx]

|  | **Univariate** | |  | **Multivariable** | |
| --- | --- | --- | --- | --- | --- |
|  | **Odds Ratio (95% CI)** | ***P*-value** |  | **Odds Ratio (95% CI)** | ***P*-value** |
| Dep: only MetS |  |  |  |  |  |
| HbA1c level (increase 1.0%) | 7.53 (6.51–8.70) | <0.001 |  | 3.38 (2.88–4.00) | <0.001 |
| Age (increase 1 year) | 1.03 (1.03–1.03) | <0.001 |  | 1.03 (1.03–1.04) | <0.001 |
| Sex (ref: male) | 0.61 (0.55–0.66) | <0.001 |  | 0.68 (0.58–0.79) | <0.001 |
| Body mass index (increase 1 kg/m^2^) | 1.39 (1.36–1.41) | <0.001 |  | 1.40 (1.37–1.42) | <0.001 |
| Alcohol intake (ref: abstinence) |  |  |  |  |  |
| Moderate drinking | 0.87 (0.78–0.97) | 0.009 |  | 1.10 (0.96–1.25) | 0.168 |
| Heavy drinking | 1.88 (1.53–2.31) | <0.001 |  | 2.46 (1.91–3.17) | <0.001 |
| Smoking status (ref: non-smoker) |  |  |  |  |  |
| Ex-smoker | 1.58 (1.41–1.78) | <0.001 |  | 1.03 (0.87–1.23) | 0.781 |
| Current smoker | 1.65 (1.47–1.85) | <0.001 |  | 1.33 (1.12–1.58) | 0.001 |
| Physical activity (ref: no) | 0.82 (0.74–0.90) | <0.001 |  | 0.83 (0.74–0.92) | 0.001 |
| Dep: only CKD |  |  |  |  |  |
| HbA1c level (increase 1.0%) | 9.32 (6.16–14.11) | <0.001 |  | 2.13 (1.33–3.40) | 0.002 |
| Age (increase 1 year) | 1.12 (1.11–1.14) | <0.001 |  | 1.11 (1.09–1.13) | <0.001 |
| Sex (ref: male) | 0.67 (0.51–0.87) | 0.003 |  | 0.75 (0.46–1.22) | 0.249 |
| Body mass index (increase 1 kg/m^2^) | 1.05 (1.01–1.09) | 0.023 |  | 1.05 (0.95–1.15) | 0.347 |
| Alcohol intake (ref: abstinence) |  |  |  |  |  |
| Moderate drinking | 0.35 (0.27–0.46) | <0.001 |  | 0.86 (0.62–1.18) | 0.340 |
| Heavy drinking | – | – |  | – | – |
| Smoking status (ref: non-smoker) |  |  |  |  |  |
| Ex-smoker | 2.60 (1.93–3.51) | <0.001 |  | 1.25 (0.77–2.02) | 0.360 |
| Current smoker | 0.67 (0.42–1.07) | 0.090 |  | 0.79 (0.43–1.44) | 0.434 |
| Physical activity (ref: no) | 0.83 (0.63–1.10) | 0.202 |  | 0.9 (0.71–1.31) | 0.818 |
| Waist circumference (increase 1 cm) | 1.04 (1.03–1.05) | <0.001 |  | 0.99 (0.96–1.02) | 0.645 |
| HDL cholesterol level (increase 1 mg/dL) | 0.96 (0.95–0.97) | <0.001 |  | 0.98 (0.96–0.99) | 0.003 |
| Triglyceride level (increase 1 mg/dL) | 1.00 (1.00–1.00) | 0.044 |  | 1.00 (1.00–1.00) | 0.903 |
| Systolic blood pressure (increase 1 mmHg) | 1.04 (1.04–1.05) | <0.001 |  | 1.01 (1.00–1.02) | 0.275 |
| Diastolic blood pressure (increase 1 mmHg) | 1.01 (1.00–1.02) | 0.206 |  | 1.02 (1.00–1.03) | 0.127 |
| Coronary artery disease (ref: no) | 9.45 (5.95–14.99) | <0.001 |  | 2.57 (1.55–4.26) | <0.001 |
| Cerebrovascular accident (ref: no) | 9.71 (5.86–16.08) | <0.001 |  | 2.60 (1.49–4.54) | 0.001 |
| Dep: both MetS and CKD |  |  |  |  |  |
| HbA1c level (increase 1.0%) | 21.49 (10.86–42.52) | <0.001 |  | 4.12 (1.80–9.39) | 0.001 |
| Age (increase 1 year) | 1.12 (1.10–1.15) | <0.001 |  | 1.10 (1.07–1.13) | <0.001 |
| Sex (ref: male) | 0.93 (0.60–1.43) | 0.727 |  | 1.76 (0.74–4.18) | 0.200 |
| Body mass index (increase 1 kg/m^2^) | 1.26 (1.20–1.33) | <0.001 |  | 1.11 (0.962–1.29) | 0.149 |
| Alcohol intake (ref: abstinence) |  |  |  |  |  |
| Moderate drinking | 0.34 (0.22–0.54) | <0.001 |  | 0.90 (0.52–1.57) | 0.704 |
| Heavy drinking | 0.18 (0.03–1.31) | 0.090 |  | 0.74 (0.09–6.29) | 0.778 |
| Smoking status (ref: non-smoker) |  |  |  |  |  |
| Ex-smoker | 1.67 (1.00–2.77) | 0.049 |  | 1.01 (0.42–2.42) | 0.982 |
| Current smoker | 0.70 (0.35–1.39) | 0.309 |  | 0.87 (0.31–2.45) | 0.791 |
| Physical activity (ref: no) | 0.62 (0.39–0.99) | 0.045 |  | 0.95 (0.56–1.63) | 0.863 |
| Waist circumference | 1.12 (1.10–1.14) | <0.001 |  | 1.11 (1.05–1.17) | <0.001 |
| HDL cholesterol level | 0.88 (0.86–0.90) | <0.001 |  | 0.88 (0.85–0.92) | <0.001 |
| Triglyceride level | 1.01 (1.01–1.01) | <0.001 |  | 1.01 (1.01–1.01) | <0.001 |
| Systolic blood pressure | 1.06 (1.05–1.07) | <0.001 |  | 1.06 (1.04–1.07) | <0.001 |
| Diastolic blood pressure | 1.02 (1.00–1.04) | 0.117 |  | 1.00 (0.97–1.03) | 0.857 |
| Coronary artery disease | 9.50 (4.66–19.37) | <0.001 |  | 1.76 (0.71–4.40) | 0.224 |
| Cerebrovascular accident | 9.14 (4.12–20.26) | <0.001 |  | 1.41 (0.50–3.97) | 0.519 |

*Multivariable analysis for only MetS was performed using HbA1c level, age, sex, body mass index, alcohol intake, smoking status, and physical activity. Multivariable analyses for only CKD or both MetS and CKD were as performed using HbA1c level, age, sex, body mass index, alcohol intake, smoking status, physical activity, waist circumference, HDL cholesterol level, triglyceride level, systolic blood pressure, diastolic blood pressure, coronary artery disease, and cerebrovascular accident.

Abbreviations: CI, confidence interval; Dep, dependent variable; MetS, metabolic syndrome; CKD, chronic kidney disease; HDL, high-density lipoprotein.
