# Supplementary material for: HbA1c Levels Are Associated with Chronic Kidney Disease in a Non-Diabetic Adult Population: A Nationwide Survey (KNHANES 2011–2013)
Source: PLoS One. 2015 Dec 30;10(12):e0145827. doi: 10.1371/journal.pone.0145827 (PMC4696727; doi:10.1371/journal.pone.0145827)
Supplement: S3 Table — (DOCX) [file pone.0145827.s004.docx]

| **Models** | **AUC** | ***P*-value (difference between AUCs)** | **Relative IDI** | |  | **Category-Free NRI** | |
| --- | --- | --- | --- | --- | --- | --- | --- |
|  |  |  | **Values** | ***P*-value** |  | **Values** | ***P*-value** |
| Dep: only MetS |  |  |  |  |  |  |  |
| Multivariable model without HbA1c | 0.82810 | Ref | – | – |  | – | – |
| Multivariable model with HbA1c | 0.83879 | <0.0001 | 0.11109 | <0.0001 |  | 0.37141 | <0.0001 |
| Dep: only CKD |  |  |  |  |  |  |  |
| Multivariable model without HbA1c | 0.89970 | Ref | – | – |  | – | – |
| Multivariable model with HbA1c | 0.90413 | 0.02 | 0.096432 | 0.0011 |  | 0.071110 | 0.0008 |
| Dep: both MetS and CKD |  |  |  |  |  |  |  |
| Multivariable model without HbA1c | 0.96933 | Ref | – | – |  | – | – |
| Multivariable model with HbA1c | 0.97218 | 0.1283 | 0.11675 | 0.0014 |  | 0.64956 | <0.0001 |

*Multivariable model for only MetS was performed using age, sex, body mass index, alcohol intake, smoking status, and physical activity. Multivariable models for only CKD or both MetS and CKD were as performed using age, sex, body mass index, alcohol intake, smoking status, physical activity, waist circumference, HDL cholesterol level, triglyceride level, systolic blood pressure, diastolic blood pressure, coronary artery disease, and cerebrovascular accident.

Abbreviations: AUC, area under curve; IDI, integrated discrimination improvement; NRI, net reclassification improvement; Dep, dependent variable ; MetS, metabolic syndrome ;HbA1c, glycated hemoglobin A1c; CKD, chronic kidney disease.
